# Influence of percutaneous catheter intervention for congenital perimembranous ventricular septal defects in children on the cardiac conduction system and associated risk factors: a meta-analysis

**DOI:** 10.1186/s13019-022-01751-8

**Published:** 2022-02-16

**Authors:** Yu-Qing Lei, Wen-Hao Lin, Shi-Hao Lin, Wen-Peng Xie, Jian-Feng Liu, Qiang Chen, Hua Cao

**Affiliations:** 1 Department of Cardiac Surgery, Fujian Branch of Shanghai Children’s Medical Center, Fuzhou, China; 2 Fujian Children’s Hospital, Fuzhou, China; 3 Fujian Maternity and Child Health Hospital, Affiliated Hospital of Fujian Medical University, Fuzhou, China; 4 Fujian Key Laboratory of Women and Children’s Critical Diseases Research, Fujian Maternity and Child Health Hospital, Fuzhou, China

**Keywords:** Perimembranous ventricular septal defects, Pediatric patients, Transcatheter therapy, Arrhythmias, Risk factors, Meta-analysis

## Abstract

**Background:**

The aim of this study was to investigate adverse outcomes and risk factors for the cardiac conduction system in children with perimembranous ventricular septal defects (pmVSDs) who had been treated by catheter intervention.

**Method:**

PubMed, EMBASE, Web of Science, and the Cochrane Library were searched for studies in English on interventional treatment of pmVSDs in pediatric patients published up to the end of October 15, 2020. We used random- or fixed-effect models to obtain pooled estimates of the success rate and postoperative complications.

**Results:**

A total of 1650 pediatric patients from 8 publications were included, with a mean age ranging from 3.44 to 8.67 years old. The pooled estimate of successful implantation was 98.2% (95% CI 97.1–99.4%, I^2^ = 69.4%; *P* < 0.001), and the incidence of cardiac conduction system complications was 17.4% (95% CI 8.4–26.4%, I^2^ = 96.1%; *P* < 0.001), among which the incidence of heart block was 14.8% (95% CI 6.4–23.3%, I^2^ = 96.9%; *P* = 0.001). The incidence of impulse formation disorders was 4.1% (95% CI 0.7–7.6%, I^2^ = 91.7%; *P* = 0.019), and the incidence of complete atrioventricular block was 0.8% (95% CI 0.3–13%, I^2^ = 0.0%; *P* = 0.001). Risk factors for newly emerging arrhythmias included the VSD size MD = 0.89 (95% CI 0.46–1.32, I^2^ = 0%; *P* < 0.0001) and device size MD = 1.26 (95% CI 0.78–1.73, I^2^ = 0%; *P* < 0.00001).

**Conclusions:**

Percutaneous catheter intervention is safe and effective in treating pediatric patients with pmVSD, and the risk factors leading to arrhythmias include the sizes of the pmVSD and device.

## Introduction

Ventricular septal defects (VSDs) are the most common congenital heart disease, accounting for 20–30% of all congenital heart malformations. This disease can be categorized according to the defective part, the perimembranous VSD (pmVSD) being the most common with an incidence of 70–80% [1, 2]. Open-heart surgical repair with median sternotomy under cardiopulmonary bypass (CPB) has been the primary approach to treat pmVSD for many years. However, traditional surgical treatment is associated with many problems, such as major trauma, long postoperative recovery, and surgical incision scarring, that cause physical and mental harm to patients, especially children [3, 4]. In 1988, Lock and his colleagues first reported successful catheterization using the Rashkind double-umbrella device in seven patients with VSDs [5]. In 1999, Amplatzer muscular VSD (AGA Medical Corp, Golden Valley, Minnesota) was used in the clinical treatment of muscular VSD. [6] The treatment was subsequently widely popularized and applied. As the anatomic site of pmVSDs is close to the cardiac conduction system, there is an unacceptably high risk of early and late complete heart block and other types of arrhythmias during the therapeutic process. [7, 8] Percutaneous catheter therapy offers the advantages of minimal invasiveness, shorter postoperative recovery, and no thoracotomy scars and therefore has been widely used in China, India, and other countries [9, 10]. Parents of patients are concerned about thoracotomy and the resulting scars, as well as complications following cardiopulmonary bypass, and are therefore more likely to prefer a less invasive surgical approach. However, interventional closure is also associated with complications, such as arrhythmia, valve injury, residual shunt, and hemolysis, and can only partially replace surgical treatment. Accurately identifying relevant risk factors and preventing postoperative complications has become clinically urgent.

A meta-analysis was previously carried out to compare the safety and efficacy of different treatments for pmVSDs. However, there is a dearth of meta-analyses on the influence of percutaneous catheter intervention in children with pmVSDs on the cardiac conduction system. In this study, the incidence and prognosis of interventional-therapy-related complications in children with pmVSD are investigated, and the risk factors for this treatment are analyzed using cardiac electrophysiology to provide medical evidence for the utility of this intervention in clinical diagnosis and treatment.

## Methods

### Literature search strategy

A systematic search of the English literature was performed in PubMed, EMBASE, Web of Science, and the Cochrane Library by two independent researchers (Wen-Peng Xie and Jian-Feng Liu) from the inception to the end (October 15, 2020) of the study. The retrieval keywords included perimembranous VSD, interventional closure and pediatric patients. The search strategy was (heart septal defects, ventricular [MeSH Terms] OR ventricular septal defect, perimembranous [Title/Abstract] OR intraventricular septal defect, perimembranous [Title/Abstract]) AND (transcatheter closure [Title/Abstract] OR intervention [Title/Abstract] OR interventional therapy [Title/Abstract] OR percutaneous [Title/Abstract]). References of retrieved articles and reviews were also manually screened to obtain relevant eligible studies. Any disagreements were resolved through third-party discussion or consultation.

### Study selection and quality assessment

**Fig. 1:**
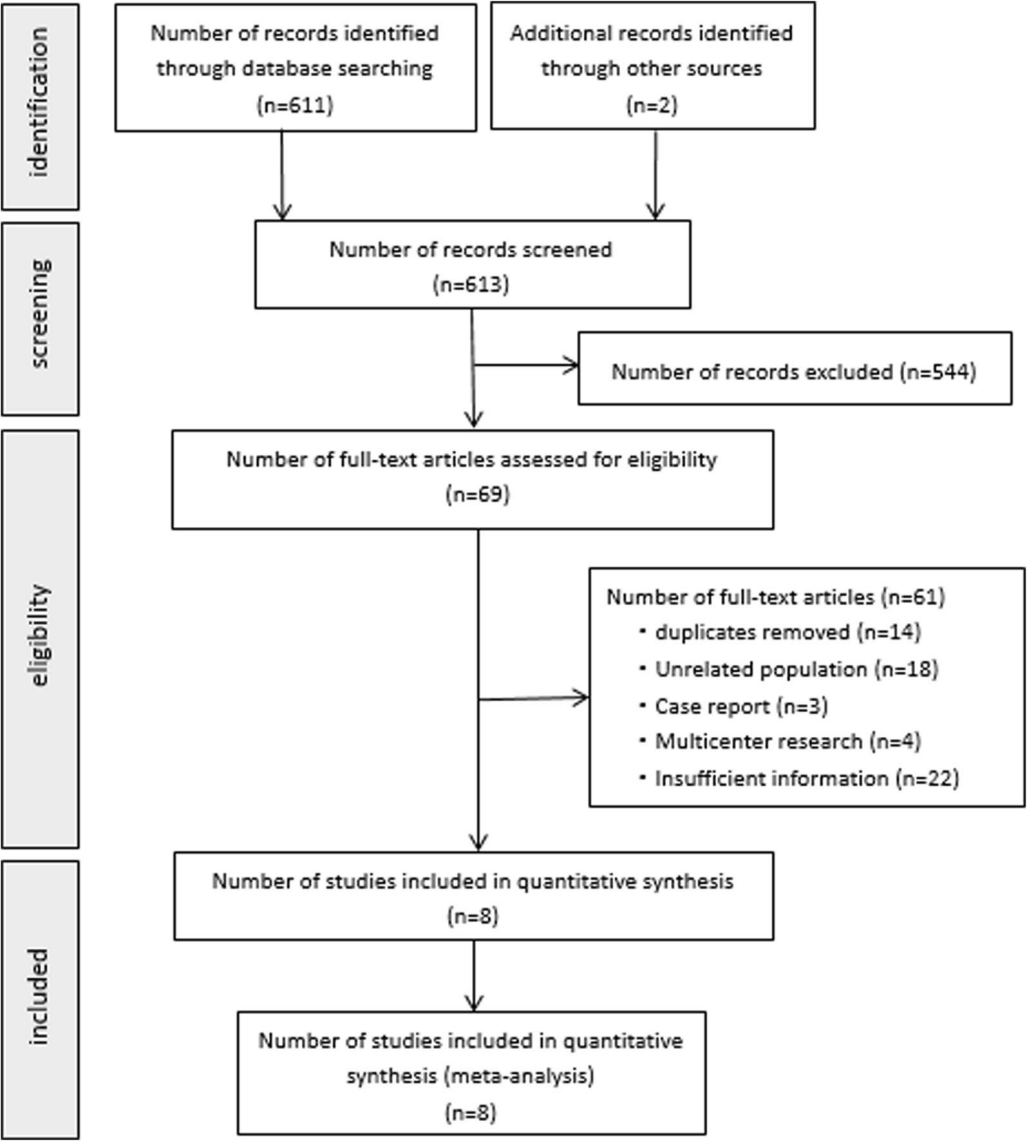
Flow chart of study selection

The following inclusion criteria were preestablished: (1) studies (randomized and nonrandomized) reporting percutaneous catheter intervention for pmVSDs in children (younger than 18 years old); (2) inclusion of only the most recent results if several studies were performed at the same center; and (3) no contraindications to interventional therapy in all cases. Exclusion criteria included (1) animal experiments, multicenter studies, and case reports; (2) case series studies with a sample size of less than ten for which valid data could not be provided; and (3) studies on patients with secondary VSD or older than 18 years. A total of 613 articles were identified in the search, of which 605 were excluded (Fig. 1). A total of 8 articles were finally included and further analyzed. [11–18].

This meta-analysis included 1 case–control study and 7 case series. We used the Newcastle–Ottawa Scale (NOS) to assess the quality of the case–control study. The NOS was used to assess the quality of studies based on the selection of cases and controls (0–4 stars), comparability of the cases and controls (0–2 stars), and the ascertainment of exposure (0–4 stars). NOS scores > 6 stars were considered to indicate high quality. [19].

We chose the quality assessment tool launched by the Australia Joanna Briggs Institute (JBI) evidence-based health care center to evaluate the quality of the case series. The evaluation tool included ten items to assess several aspects of quality, including case selection, disease or health assessment, and case information. Each item was scored as "Yes" (full description, 2 points), "No" (noncompliance, 0 points), or "Unclear" (mentioned but not described in detail, 1 point), and a final score above 70% of the total score (14 points) was considered high quality [20]. Disagreements on quality assessment were resolved through discussion.

### Data extraction

The following data were extracted by two independent authors (Wen-Peng Xie and Jian-Feng Liu) and entered into an EXCEL file: the name of the first author, year of publication, country, sample size, weight, age, sex (male/female), pmVSD size, device size, success rate, operative time, complications (including arrhythmias, valve regurgitation, and residual shunt) and number of years of follow-up. Successful closure was defined as follows: (1) no residual shunt and (2) no severe complications (including death, transfer to surgery, or reoperation.).

### Statistical analysis

STATA 16.0 (STATA Corporation, College Station, Texas, USA) and Review Manager version 5.4.1 (The Nordic Cochrane Centre, Copenhagen, Denmark) were used to pool estimates. For the case series and case–control studies, dichotomous variables were represented by odds ratios (ORs) and 95% CIs, whereas continuous variables were represented by standard mean differences (SMDs) and 95% CIs. The extent of heterogeneity across studies was evaluated using the inconsistency statistic (I^2^) and the Q test. A fixed-effect model was used when I^2^ < 50% or *P* ≥ 0.1 indicated statistical homogeneity between studies. However, I^2^ ≥ 50% or *P* < 0.1 was considered to indicate substantial heterogeneity, and a random-effect model was adopted [21, 22]. Based on the Cochrane Handbook, publication bias could not be accurately assessed because a limited number of studies (below 10) were included.

## Results

A total of 1650 pediatric patients from 8 studies (Table 1) were further analyzed. The median follow-up duration was 3–78 months, and the average age of the children was 3.44–8.67 years old.

**Table 1:** Characteristic details

First author	Years	Study type	Study design	Country	Arrhythmias pts(n)	Total pts(n)	Quality assessment
Mehdi Ghaderian	2015	Case series	RS	Iran	11	110	15 points
Yifei Li	2015	Case series	RS	China	103	553	15 points
Hao Li	2019	Case series	RS	China	37	253	17 points
Krishna D. Mandal	2018	Case series	RS	China	9	186	16 points
Dragos Predescu	2008	Case series	RS	Canada	4	20	14 points
Jayal Hasmukhbhai Shah	2020	Case series	RS	India	35	376	16 points
B D Thanopoulos	2003	Case series	RS	Greece	3	10	13 points
Rong Yang	2010	Case–control	RS	China	22	142	7 stars*

^*^Use the Newcastle–Ottawa scale

**Fig. 2:**
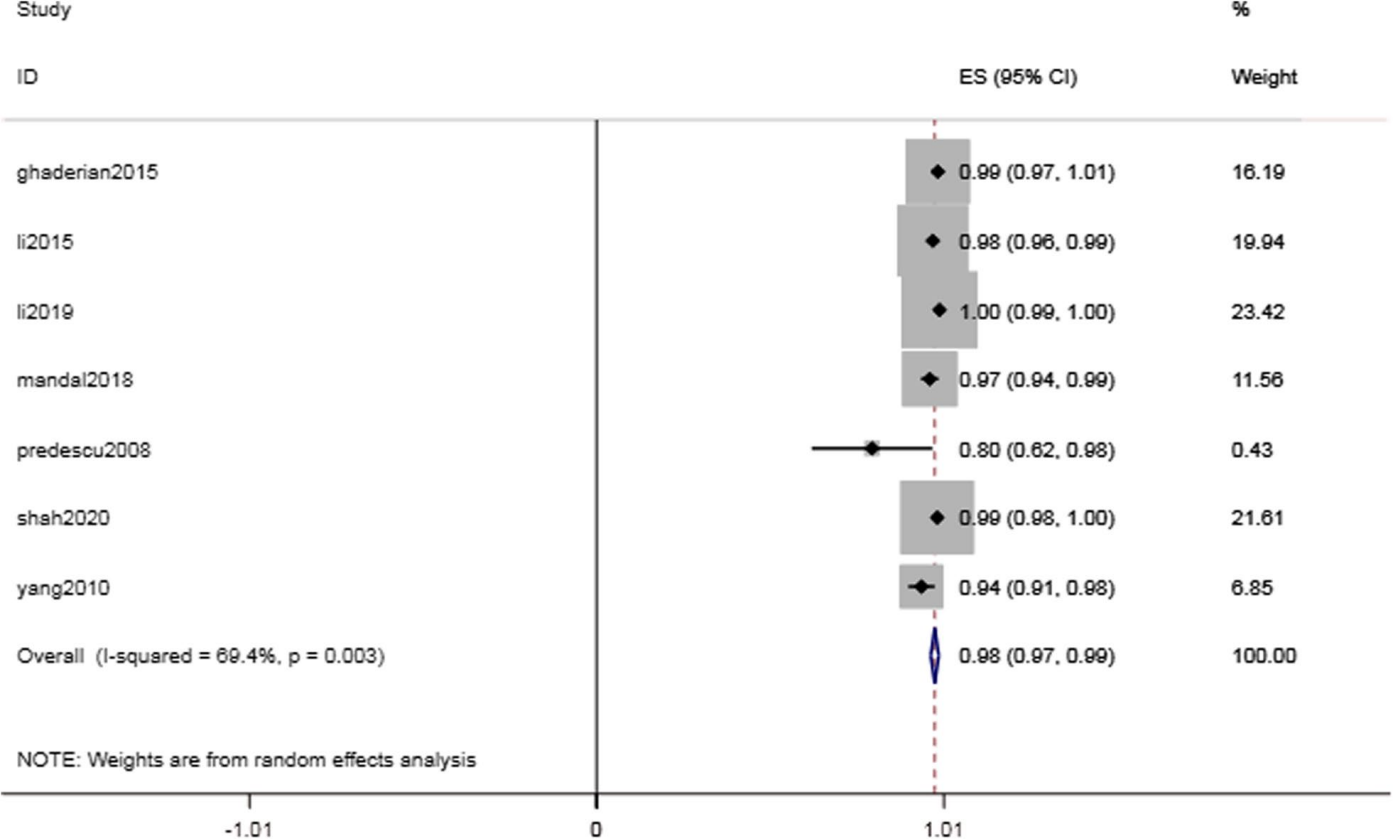
Forest plot of success rate

The success rate of interventional catheterization in pediatric patients was high. A success rate of 100% was reported in Thanopoulos’ study, and a success rate greater than 90% was reported in six out of the remaining seven studies. The Q statistic showed significant heterogeneity (I^2^ = 82.1%). The results of a sensitivity analysis conducted by excluding one study at a time showed the largest heterogeneity came from Thanopoulos’ study. Although heterogeneity still existed after eliminating this study, there was no significant difference in the success rate between the random- and fixed-effect models, MD = 98.2% (95% CI 97.1–99.4%, I^2^ = 69.4%; *P* < 0.001) (Fig. 2).

**Fig. 3:**
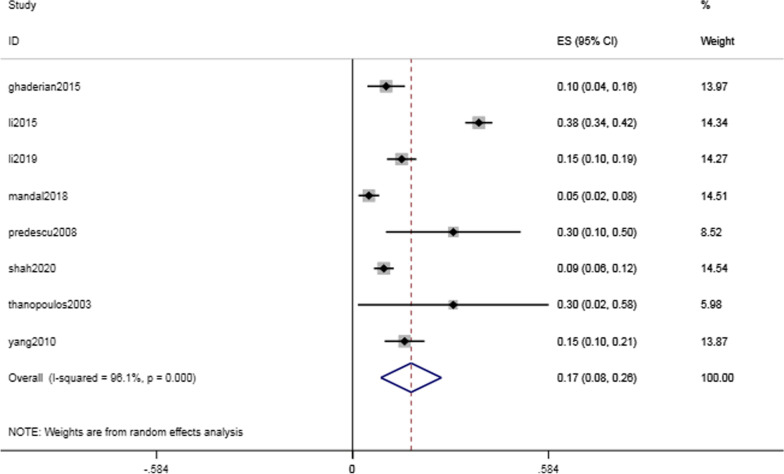
Forest plot of cardiac conduction system complications

**Fig. 4:**
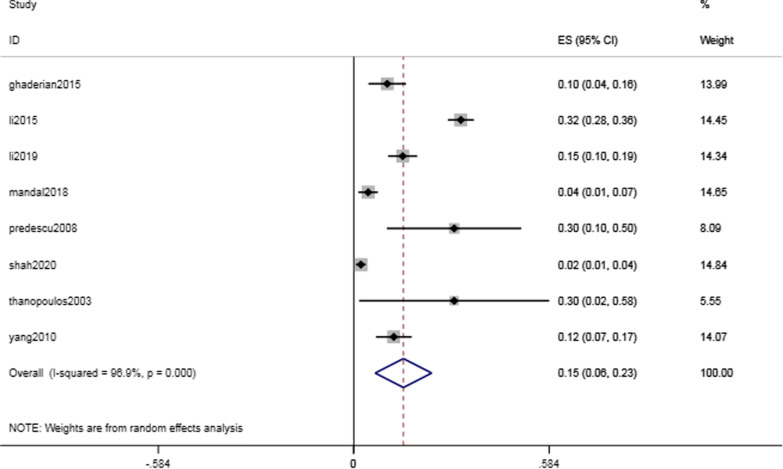
Forest plot of heart block

**Fig. 5:**
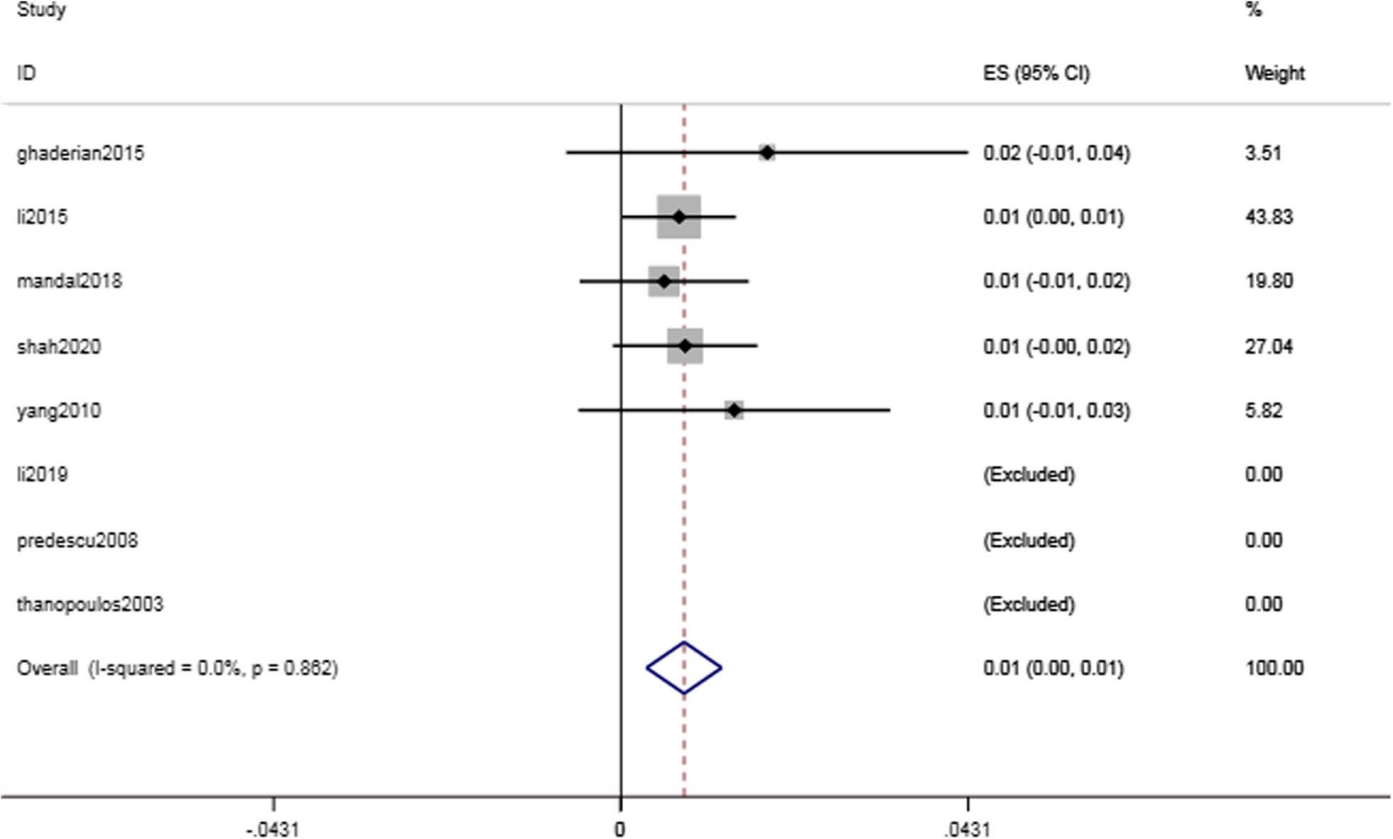
Forest plot of complete atrioventricular block

**Fig. 6:**
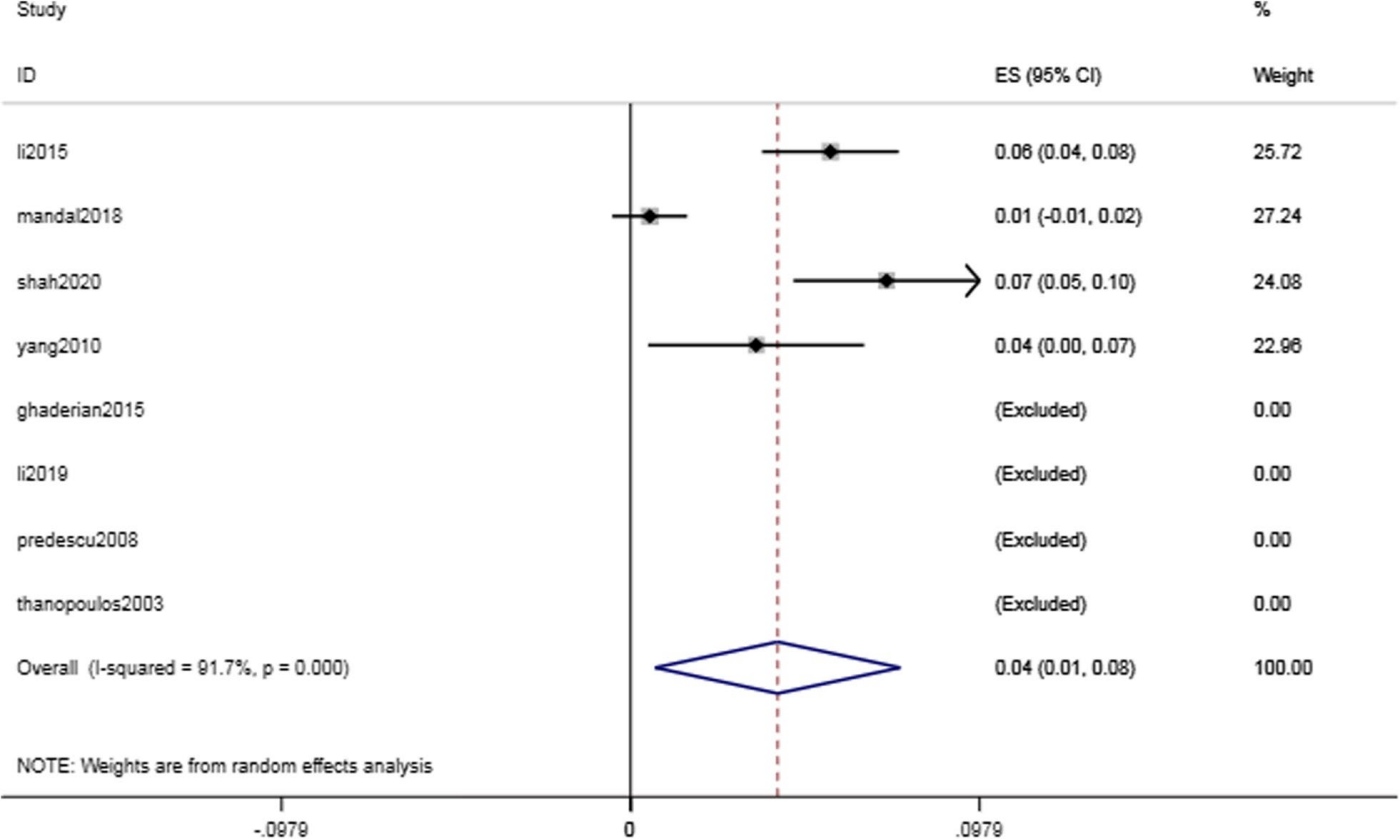
Forest plot of impulse formation disorders

Complications of the cardiac conduction system were common but critical, with a rate of 17.4% (95% CI 8.4–26.4%, I^2^ = 96.1%; *P* < 0.001) (Fig. 3). The intraoperative and postoperative conduction abnormalities were categorized into heart block, such as atrioventricular block, and heartbeat disorder, such as nonparoxysmal atrioventricular intersectional tachycardia. The incidence of heart block was 14.8% (95% CI 6.4–23.3%, I^2^ = 96.9%; *P* = 0.001) (Fig. 4). However, as shown in Table 2, the majority of the abnormalities were temporary arrhythmias, and sinus rhythm could be restored after intraoperative surgical suspension or postoperative corticosteroid treatment. The rate of complete atrioventricular block (cAVB) was 0.8% (95% CI 0.3–13%, I^2^ = 0.0%; *P* = 0.001) (Fig. 5), and the incidence rate of impulse disorder was 4.1% (95% CI 0.7–7.6%, I^2^ = 91.7%; *P* = 0.019) (Fig. 6). Due to the severity of cAVB, we have listed all patients for whom cAVB was reported in the included studies and the corresponding treatment and outcomes in Table 3.

**Table 2:** Cardiac conduction system complications

Study	Arrhythmias pts(n)	Heart block pts(n)	Impulse formation disorders pts(n)	Transient pts(n)	cAVB pts(n)
ghaderian2015	11	11	0	9	2
li2015	208	177	31	175	4
li2019	37	37	0	37	0
mandal2018	9	8	1	6	1
predescu2008	6	6	0	3	0
shah2020	35	8	27	34	3
thanopoulos2003	3	3	0	3	0
yang2010	22	17	5	22	2

**Table 3:** Treatment and Outcomes of Patients with cAVB

Study	Total pts(n)	cAVB pts(n)	Features	Treatment			Outcomes
				Transferred to surgery	*Comprehensive symptomatic treatment	Permanent pacemaker	
ghaderian2015	110	2	intraoperative cAVB	√			Sinus rhythm
			Late-onset cAVB (2 weeks)			√	Sinus rhythm
li2015	553	4	All were late-onset cAVB (1 months)		√		Sinus rhythm
li2019	253	0	-	–	–	–	–
mandal2018	186	1	Late-onset cAVB (4 days)		√		Sinus rhythm
predescu2008	20	0	–	–	–	–	–
shah2020	376	3	1 case was intraoperative cAVB	√			NM
			2 cases were postoperative cAVB		√		NM
thanopoulos2003	10	0	-	–	–	–	–
yang2010	142	2	Both were postoperative cAVB		√		Sinus rhythm

^*^Comprehensive symptomatic treatment: steroid therapy and implanting temporary pacemakers

NM: not mentioned

**Fig. 7:**
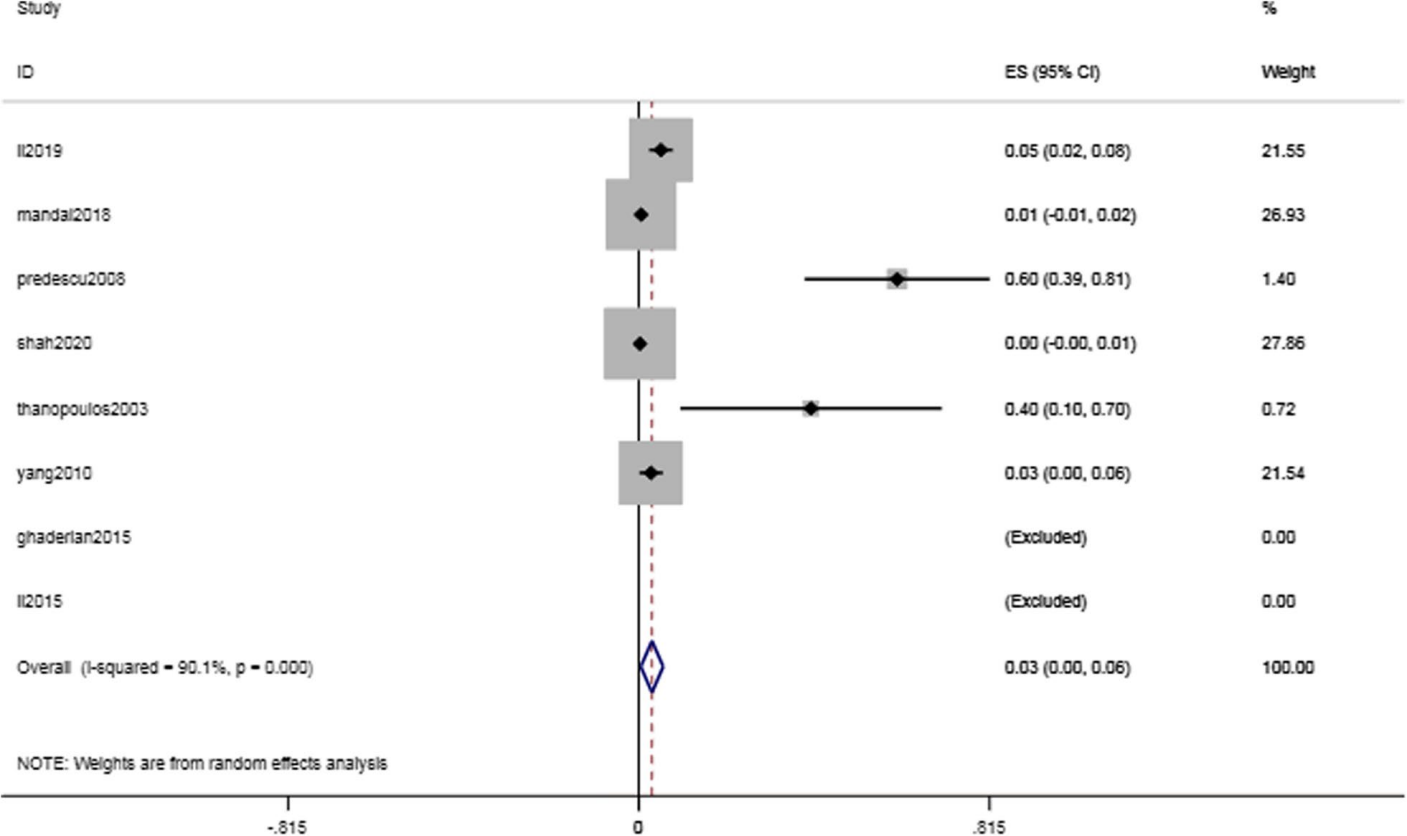
Forest plot of valve regurgitation

Postoperative valve regurgitation was relatively low in pediatric patients undergoing transcatheter device closure of VSDs. Six out of eight studies reported the occurrence of postoperative valve regurgitation. The valve regurgitation rate was found to be 3.1% (95% CI 0.5–5.7%, I^2^ = 90.1%; *P* = 0.021) (Fig. 7).

**Fig. 8:**
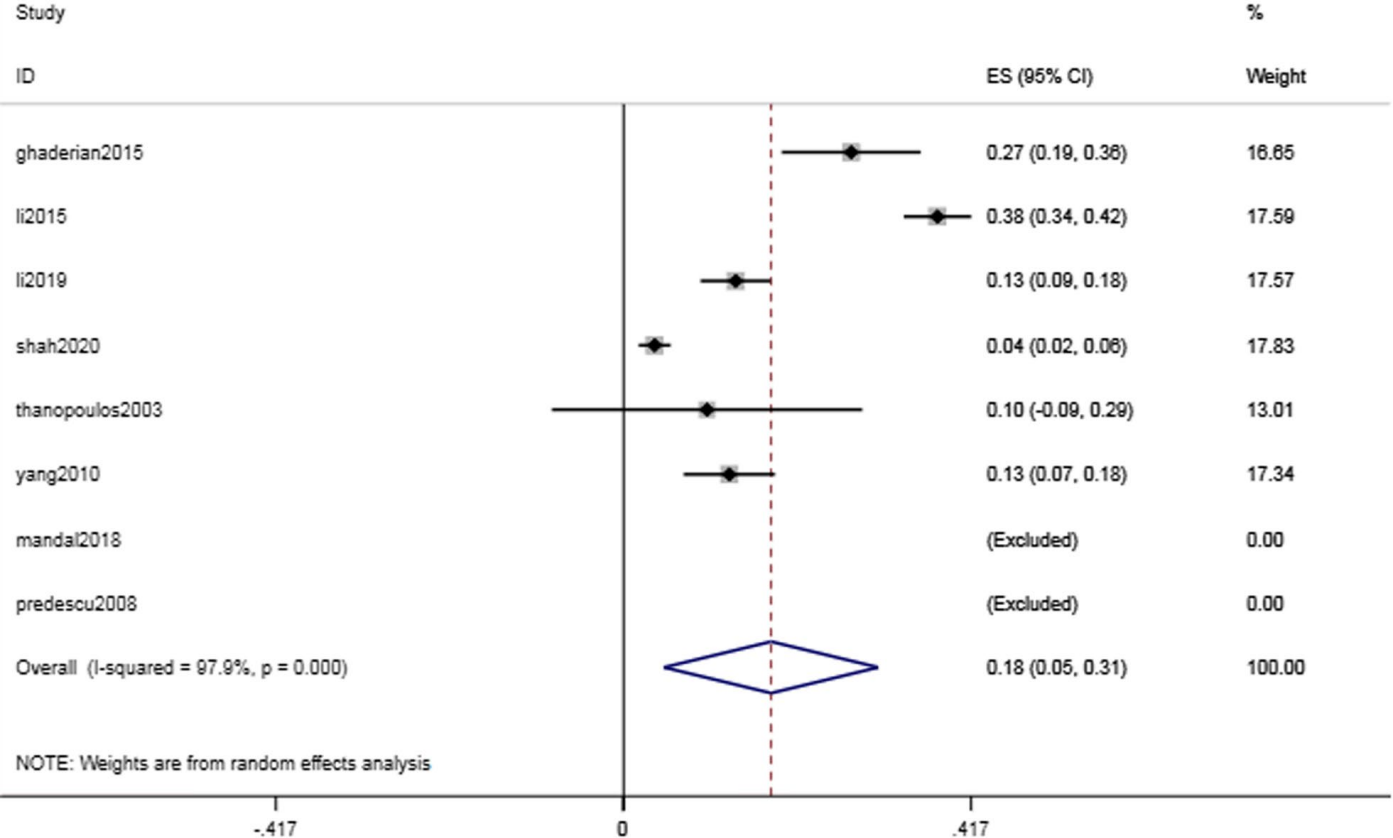
Forest plot of the immediate residual shunt

Among all the included studies, only six reported the number of patients with immediate residual shunts after intervention. The immediate residual shunt rate was found to be 17.7% (95% CI 4.9–30.5%, I^2^ = 97.9%; *P* = 0.007) (Fig. 8), and the residual shunt rate at the end of follow-up was found to be 1.2% (95% CI 0.1–2.4%, I2 = 36.7%; *P* = 0.069).

No deaths, but a minimal number of occasional adverse events, were reported in the included studies. There was one case of intraoperative device embolization [16]. Hematuria occurred in two cases [13, 16], device prolapse occurred in four patients, [15] and hemolysis occurred in seven patients [13–15]. Three patients bled at the puncture site, and three patients had a groin hematoma [14]. No other adverse events, such as endocarditis or aortic rupture, occurred.

**Fig. 9:**
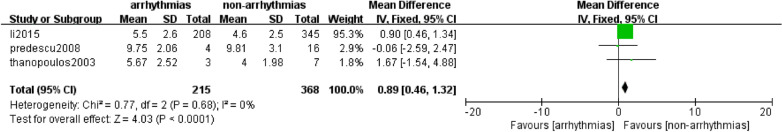
Forest plot of the relationship between the size of pmVSD defects and arrhythmias

**Fig. 10:**
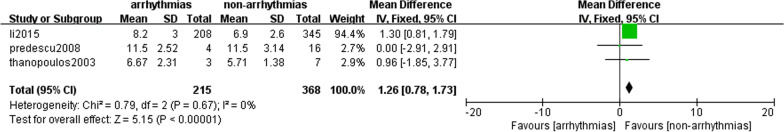
Forest plot of the relationship between the size of occluders and arrhythmias

We used the raw data of three studies to analyze the risk factors (age, VSD defect size, and occluder size) leading to intraoperative and postoperative arrhythmias in children [12, 15, 17]. We categorized the patients into a newly emerging arrhythmia group and a nonarrhythmia group. The average age for both groups was MD = − 2.07 (95% CI − 4.52 to 0.37, I^2^ = 79%; *P* = 0.10). The comparison result for the VSD defect size was MD = 0.89(95%CI: 0.46–1.32, I^2^ = 0%; *P* < 0.0001) (Fig. 9), and the comparison result for the occluder size was MD = 1.26 (95%CI: 0.78–1.73, I^2^ = 0%; *P* < 0.00001) (Fig. 10).

## Discussion

Previous studies have indicated a high incidence of cAVB from transcatheter closure using the Amplatzer occluder, ranging from 3 to 20% during early and mid-term follow-up periods [8, 23]. The continuous accumulation of clinical experience in interventional therapy and technical improvements in occluders have resulted in a reduction in the incidence of cAVB in recent years. Nevertheless, the possibility of various types of conduction abnormalities has been reported in some clinical studies. Compared with traditional surgical therapy and minimally invasive surgical treatment, interventional catheterization for patients with pmVSD has many advantages and is widely used in clinical practice. Nevertheless, complications, especially arrhythmia, have been found to be more likely to occur when performing the intervention on children. Consequently, we collected and analyzed studies on catheter interventional treatment for pediatric patients with pmVSD. Our aim in reviewing the safety and efficacy of this treatment in children was to provide a preoperative assessment of potential risk factors to predict, reduce, or prevent adverse events in the cardiac conduction system.

For a total of 1650 pediatric patients from 8 studies, we mainly analyzed the success rate of interventional therapy, common complications, and risk factors for cardiac conduction abnormalities in children. In all the included studies, there were no patient deaths and a zero incidence rate of severe complications, such as aortic rupture. However, intervention was stopped for one patient due to intraoperative equipment embolization [16]. There were 2 cases of hematuria. One case was severe and improved after surgical repair, whereas the other case was mild and improved after symptomatic treatment [13, 16]. The condition of four patients with occluder prolapse after the first intervention improved after large occluders were replaced. [15] Patients with hemolysis and bleeding recovered after symptomatic treatment. Severe cases improved after blood transfusion, and mild cases improved after symptomatic treatment [13–15]. A 100% success rate was reported in one study [17], and when we excluded this source of heterogeneity, the pooled success rate was 98.2% (95% CI 97.1–99.4%, I^2^ = 69.4%; *P* < 0.001), indicating that transcatheter treatment was safe and feasible.

The incidence of immediate postoperative arrhythmias was 17.4% (95% CI 8.4–26.4%, I^2^ = 96.1%; *P* < 0.001). We further classified arrhythmias as heart block and heartbeat disorder, with incidences of 14.8% (95% CI 6.4–23.3%, I^2^ = 96.9%; *P* = 0.001) and 4.1% (95% CI 0.7–7.6%, I^2^ = 91.7%; *P* = 0.019), respectively. The most serious complication, cAVB, had an incidence of 0.8% (95% CI 0.3–13%, I^2^ = 0.0%; *P* = 0.001). Table 3 shows the estimated incidence of cAVB combined with patient-specific characteristics. We found that most arrhythmias were transient, that is, sinus rhythm could be restored by discontinuing surgery, steroid therapy, and even implanting temporary pacemakers in more severe cases. Studies have shown that young patients have a myocardium with a high water content and soft structure. Myocardial edema caused by friction against the guide wire or occluder has resulted in a high incidence of cAVB. [24].

As the anatomical site of pmVSD is close to the tricuspid and aortic valves, valve injury caused by catheter intervention should be considered. However, the pooled incidence of valve regurgitation was 3.1% (95% CI 0.5–5.7%, I^2^ = 90.1%; *P* = 0.021), and most cases were mild to moderate, with gradual improvement during the follow-up period. Among these patients, 2 had clear valve regurgitation after occluder insertion and were finally treated by surgery. Valve regurgitation is associated with using an oversized occluder or an insufficient distance between the occluder edge and the valve. A diameter of the left ventricular disc of the occluder exceeding 50% of the circumference of the inferior aortic outflow tract has been reported to lead to outflow tract deformation and valve dysfunction [25]. A total of 13 patients had reflux before operation. These patients were continuously monitored after operation, followed up for 2 years, and their condition eventually improved. The remaining cases were onset patients with mild valve regurgitation evaluated by transthoracic echocardiography (TTE). The regurgitation disappeared without treatment during follow-up. The regurgitation may have resulted from the impact of the occluder on the aortic lobule, resulting in immediate AR and interference with the tendon, resulting in TR. [26–28] Therefore, echocardiography is crucial for preoperative, intraoperative and postoperative monitoring. The position and distance between the valve and the defect should be carefully evaluated before the operation. Care should be taken to prevent valve damage during surgery. Postoperative follow-up should be carried out on schedule to find and solve problems in a timely manner.

The pooled incidence of the most common postoperative complication, residual shunt, was 17.7% (95% CI 4.9–30.5%, I^2^ = 97.9%; *P* = 0.007) immediately after catheter intervention. During the follow-up, the residual shunt disappeared gradually without treatment. The final pooled residual shunt incidence was 1.2% (95% CI 0.1–2.4%, I2 = 36.7%; *P* = 0.069), which indicated that most residual shunts were self-limiting.

We further analyzed risk factors for heart block using limited original data in terms of the sizes of the VSD and device. Among the 8 included studies, 5 reported the age of children (the minimum age was 1.5–2.5 years), and 2 reported the children's weight (the minimum weight was 9–10 kg). Arrhythmia was found to be related to weight and age in a large sample of patients with VSD [29]. However, as age and weight were not consistently reported in the 8 included studies, these two aspects were not considered in our meta-analysis. The VSD size MD = 0.89 (95% CI 0.46–1.32, I^2^ = 0%; *P* < 0.0001) and the defect size MD = 1.26 (95% CI 0.78–1.73, I^2^ = 0%; *P* < 0.00001) were associated with cardiac conduction abnormalities. These results were consistent with those of previous studies [30, 31]. Notably, the forest plots show that the diameter of the pmVSDs and occluders for postoperative arrhythmia patients were higher than those for nonarrhythmia patients. Indications must be strictly controlled before intervention, and an occluder appropriate to the pmVSD size should be selected.

Transcatheter intervention for pmVSD is a promising treatment with a high success rate and low complication rate, especially because of advantages of noninvasiveness, quick recovery, and a short hospital stay. Parents consider multiple factors comprehensively in selecting treatment for children, which is why intervention is becoming increasingly commonly for pediatric patients. Although the safety and efficacy of transcatheter closure have been demonstrated [32, 33], the incidence of cardiac conduction system complications, especially cAVB, remains a significant concern. In our meta-analysis, the incidence of cAVB was 0.8% and the risk factors for arrhythmias included the sizes of VSD defects and occluders. The anatomical features of pmVSD make it almost impossible to prevent damage to the cardiac conduction system, making preventive measures, such as appropriate device selection (in terms of the type and size), essential. Most acute atrioventricular blocks that occur during surgery result from direct mechanical damage by the catheter or device, whereas postoperative heart blocks can result from local inflammation, edema, and fibrosis caused by the occluder.^34^ Therefore, it is necessary to identify heart block in a timely manner, take measures, and continuously monitor the entire process.

This study is the first meta-analysis of cardiac conduction system complications and risk factors in children with pmVSD after interventional therapy. The methodological strengths of the present paper include (1) a comprehensive literature search with a rigorous and systematic methodology, (2) detailed data extraction, and (3) standardized quality assessment using the JBI quality assessment tool and NOS scale.

The limitations of this study are as follows: (1) the included studies spanned nearly 20 years, and the baselines of the studies were not entirely consistent, resulting in heterogeneity across studies; (2) publication bias was not assessed in this meta-analysis because the number of included studies was limited (below 10); (3) insufficient information was provided in several studies, and follow-up periods varied among studies; and (4) all patients with VSD were not included in this meta-analysis. Thus, further studies should include a larger number of cases with sufficient data to determine risk factors and provide more convincing evidence.

## Conclusion

Transcatheter intervention is safe and effective in treating pmVSDs in pediatric patients, and the risk factors leading to heart block include the sizes of pmVSDs and occluders.

## Data Availability

All data needed to evaluate the conclusions were present in the paper and available in the PubMed database.

## Abbreviations

VSD: Ventricular septal defects
pmVSD: Peri-membranous ventricular septal defect
CPB: Cardiopulmonary bypass
NOS: Newcastle–Ottawa Scale
MD: Mean difference
cAVB: Complete atrioventricular block
TTE: Transthoracic echocardiography
